# Supplementary material for: Potential of Acidithiobacillus ferrooxidans to Grow on and Bioleach Metals from Mars and Lunar Regolith Simulants under Simulated Microgravity Conditions
Source: Microorganisms. 2021 Nov 23;9(12):2416. doi: 10.3390/microorganisms9122416 (PMC8706024; doi:10.3390/microorganisms9122416)
Supplement: Supplementary file 1 [file microorganisms-09-02416-s001.zip › microorganisms-1456266-supplementary.pdf]

Potential of *Acidithiobacillus ferrooxidans* to Grow on and Bioleach Metals from Mars and Lunar Regolith Simulants under Simulated Microgravity Conditions

Anna H Kaksonen [1,2], Xiao Deng [1,3,] Christina Morris [1], Himel Nahreen Khaleque [1], Luis Zea [4] and Yosephine Gumulya [1,5]

[1] Commonwealth Scientific and Industrial Research Organisation (CSIRO), Land and Water, Floreat, WA, Australia; anna.kaksonen@csiro.au; christina.morris@csiro.au; himel.khaleque@csiro.au; yosephine.gumulya@csiro.au;

[2] School of Biomedical Sciences, University of Western Australia, Crawley, WA, Australia

[3] International Center for Materials Nanoarchitectonics, National Institute for Materials Science, Tsukuba, Ibaraki, Japan; deng.xiao@nims.go.jp

[4] BioServe Space Technologies, Department of Aerospace Engineering Sciences, University of Colorado Boulder, Boulder, Colorado, United States; Luis.Zea@colorado.edu

[5] Centre for Microbiome Research, School of Biomedical Sciences, Queensland University of Technology, Translational Research Institute, Woolloongabba, QLD, Australia; gumulya@qut.edu.au

* Correspondence: gumulya@qut.edu.au

Supplementary material

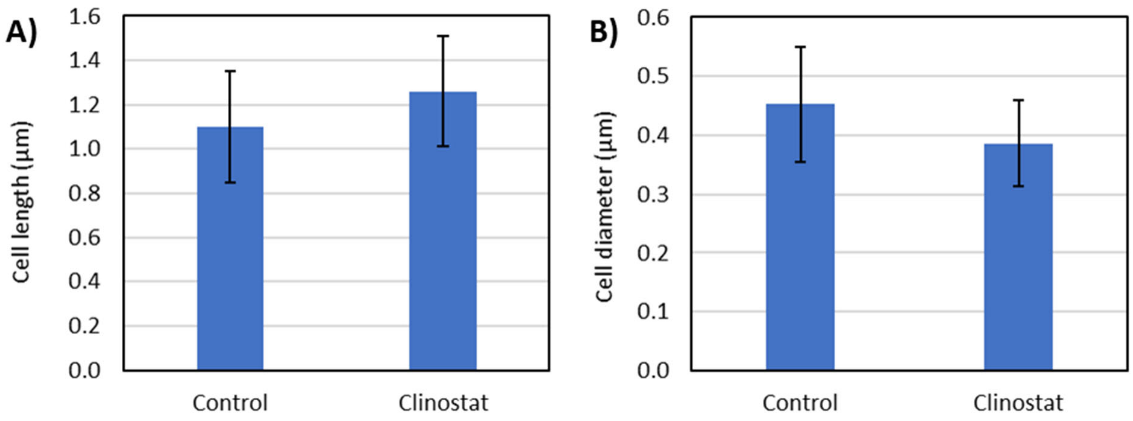

Figure S1. Cell size of *A. ferrooxidans* cultures grown under Earth gravity (control) and in simulated microgravity conditions (clinostat). Sample size ($n_{\text{anaerobic control}}$ = 9 cells, $n_{\text{anaerobic clinostat}}$ = 6 cells).
